# Comprehensive Insight into Tapetum-Mediated Pollen Development in *Arabidopsis thaliana*

**DOI:** 10.3390/cells12020247

**Published:** 2023-01-07

**Authors:** Shuaijie Wei, Ligeng Ma

**Affiliations:** College of Life Sciences, Capital Normal University, Beijing 100048, China

**Keywords:** pollen development, tapetum specification, tapetum function, pollen wall formation, secretory pathway

## Abstract

In flowering plants, pollen development is a key process that is essential for sexual reproduction and seed set. Molecular and genetic studies indicate that pollen development is coordinatedly regulated by both gametophytic and sporophytic factors. Tapetum, the somatic cell layer adjacent to the developing male meiocytes, plays an essential role during pollen development. In the early anther development stage, the tapetal cells secrete nutrients, proteins, lipids, and enzymes for microsporocytes and microspore development, while initiating programmed cell death to provide critical materials for pollen wall formation in the late stage. Therefore, disrupting tapetum specification, development, or function usually leads to serious defects in pollen development. In this review, we aim to summarize the current understanding of tapetum-mediated pollen development and illuminate the underlying molecular mechanism in *Arabidopsis thaliana*.

## 1. Introduction

In higher plants, the alternation of the life cycle mostly depends on sexual reproduction, in which normal pollen development is necessary. In the past decades, extensive studies have shown that pollen development is a complex biological process, which includes anther primordial cell differentiation, pollen mother cell meiosis, microspore mitosis, tapetal cell differentiation and programmed cell death, pollen wall formation and pollen grain release [1,2]. According to the morphological properties, anther development was divided into 14 stages by Sanders et al. [3]. Molecular analysis has demonstrated that a great number of genes are expressed and function during male reproductive development, and the proteins encoded by these genes are involved in transcriptional regulation, protein degradation, hormone biosynthesis, and signal transduction [2,4]. In addition, these studies on pollen development are also important for crop yield, crop breeding, and plant propagation.

Over the past two decades, extensive studies indicated that pollen development is coordinately controlled not only by gametophytic but also by sporophytic regulators, especially the tapetum for the latter case [5,6,7]. The tapetum is the innermost cell layer of the anther wall, which is in direct contact with the developing microsporocytes [8,9]. In general, the development of tapetal cells is initiated at floral stage 8/anther stage 4 and can be divided into three stages, including tapetum differentiation, cell binucleation, and tapetum programmed cell death [1,3]. Molecular genetic studies have discovered that tapetal cells play a vital role in pollen development by secreting nutrients, enzymes for microsporogenesis at the early stage, synthesizing and secreting callose-degrading enzymes to release microspores from tetrads, and initiating programmed cell death to provide nutrients and signals for developing microspores, multiple materials for pollen wall formation [5,7,10]. Therefore, once the tapetum develops defectively, it usually leads to serious defects in male fertility. An array of genes required for pollen development have been defined and summarized in many excellent reviews in recent years [5,6,10,11]. Here, we summarize and update the roles of tapetum in pollen development from two aspects: the specification of tapetum and its function during pollen development and further elucidate the underlying molecular mechanism in *Arabidopsis thaliana*.

## 2. The Specification of Tapetal Cells Is Essential for Pollen Development

In flowering plants, pollen development is critical for sexual reproduction and usually occurs in the special tissue: the anther. More importantly, the pollen and anther development are coordinated and precisely regulated. According to the morphological characteristics, anther development has been divided into 14 stages in *Arabidopsis* [3]. At stage 1, anther primordium emerges from the floral meristem that consists of three cell layers (L1–L3) [1,4]. Later, the outside L1 layer develops to form the epidermis of the anther by anticlinal cell division, and L3 layer cells divide and differentiate to form the connective and vascular tissues; the L2 layer cells undergo an array of divisions to form sporogenous cells and three maternal cell layers, comprising the endothecium, middle cell layer and tapetum from outside to inside [1,4,7,12].

Over the past several years, molecular and genetic studies have uncovered that the development of anthers is regulated by BCE genes of the well-known ABCE model cooperatively [6]. In *Arabidopsis*, B-class genes *APETELA3* (*AP3*) and *PISTILLATA* (*PI*), C-class gene *AGAMOUS* (*AG*), and E-class genes *SEPALLATA1/2/3/4* (*SEP1/2/3/4*) play a critical role in anther identity [4,6]. Therefore, the mutation of *AP3*, *PI*, *AG*, or *SEP1/2/3/4* usually causes serious anther developmental defects. The *SPOROCYTELESS/NOZZLE* (*SPL/NZZ*) gene encodes a MADS-box transcription factor and is expressed in the L2 layer; and it is activated by AG protein and functions in the specification of reproductive cells [13,14,15,16]. Anthers of *spl/nzz* contain primary parietal, primary sporogenous cells, and normal initiated archesporial cells, but the pollen mother cells (PMCs) cannot be formed and the tapetum development is also defective, suggesting that the SPL/NZZ is essential for early anther development [13,16]. A recent investigation revealed that the SPL/NZZ can interact with and be phosphorylated by MITOGEN-ACTIVATED PROTEIN KINASE 3 (MPK3) and MPK6, which enhance the stability of SPL/NZZ [17]. In addition, the *BARELY ANY MERISTEM 1* and *2* (*BAM1* and *BAM2*), which encode two homologous and functional redundancy leucine-rich repeat receptor-like protein kinases (LRR-RLKs), are reported to interact with and suppress the expression of SPL/NZZ [18]. In *bam1bam2* anther, the cell division and specification of L2 derived are disordered, which leads to a lack of the endothecium, middle layer, and tapetum, in contrast to generating excess pollen mother-like cells [18]. Similar to BAM1/2, anther LRR-RLK, RECEPTOR-LIKE PROTEIN KINASE2 (RPK2) also plays an important role in anther lobe identity and early anther cell specification [19]. Owing to the disordered cell differentiation, anthers in *rpk2* lack the middle layer and exhibit hypertrophic tapetum and defective endothecium, which ultimately results in the males being sterile [19,20].

It is widely accepted that the ligand binding of RLKs usually functions in signal transduction by recruiting other RLKs as co-receptors [21]. A recent report has shown that a group of novel RLK proteins CLAVATA3 INSENSITIVE RECEPTOR KINIASEs (CIKs) functions as coreceptors of RPK2 and BAM1/2 to participate in the regulation of archesporial cell division and parietal cell specification in early anther development [20]. The anther phenotypes of these *CIKs* gene mutants are similar to the *bam1bam2* and *rpk2*, including a lack of one to three parietal cell layers and excess microsporocytes [20]. In short, this evidence revealed that CIKs function with BAM1/2 and RPK2 in the same pathway during the early anther development, but the ligand signals and substrates of the BAM1/2-CIK and RPK2–CIK complex remain to be explored in the future.

Tapetum is the most inner somatic cell layer of the anther and is adjacent to the developing microsporocyte and/or microspores directly [12]. More importantly, during the anther development, the tapetal cells can provide abundant nutrition, lipide, and enzymes for gametogenesis by the way of secreting or programmed cell death [5]. Defects in tapetum specification and development result in failed pollen development and impaired fertility [22,23,24]. Molecular genetic studies have revealed that the regulation mechanism of tapetal specification and development is a complex network [12,25].

Over the past two decades, several studies suggested that the specification of the tapetum is mainly regulated by the TPD-EMS1-SERK1/2 signaling pathway in early anther development [23,26,27,28,29,30,31,32]. The *EXCESS MICROSPOROCYTES1* (*EMS1*), which is also named *EXTRA SPOROGENOUS CDLLS* (*EXS*), encodes an LRR-RLK and is expressed in both sporogenous and parietal cells in early anther, especially, strongly expressed in tapetum, suggesting that EMS1/EXS is associated with microsporocytes and tapetal cells differentiation [26,27]. When the function of EMS1 is disrupted, the anther has no tapetal layer, but instead presents excess microsporocytes [26,27]. Cytological observation showed that these excess microsporocytes can undergo normal meiotic nuclear division, but cytokinesis fails to occur, leading to unsuccessful microsporogenesis and male sterility [27]. As an LRR-RLK, EMS1 kinase activity relies on its autophosphorylation status [30]. A recent study uncovered that EMS1 can interact with and be transphosphorylated by SOMATIC EMBRYOGENESIS RECEPTOR-LIKE KINASE1 (SERK1), which is fundamental for enhancing EMS1 kinase activity [32]. The *SERK1* also encodes an LRR-RLK protein and redundantly regulates the tapetum development with its homologous protein SERK2 [28,29]. The SERK1 and SERK2 share 90% identity in the primary amino acid sequence; therefore, neither the *serk1* nor *serk2* display obvious anther defects [28,29]. Similar to the *ems1*, the anthers of *serk1 serk2* lack the tapetal layer and produce more pollen mother cells, which suggests that SERK1/2 function with EMS1/EXS in a common pathway [28,29,32].

In addition, the *TAPETUM DETERMINANT1* (*TPD1*) was proven to function in tapetum specification, and indistinguishable phenotype compared with *ems1* and *serk1serk2* was observed in *tpd1* in early anther development [23]. *TPD1* encodes a small cysteine-rich protein with 176 amino acids, which is secreted from reproductive cells [23,31]. Furthermore, molecular studies showed that TPD1 works as the ligand to interact with tapetum precursor plasma membrane localized-EMS1 to activate EMS1 phosphorylation and then determine the tapetal cells’ fate [23,30,31,33]. Taken together, the tapetum specification is regulated by the TPD1-EMS1-SERK1/2 signal pathway, in which the TPD1 is the ligand, which is recognized by EMS1, and SERK1/2 may be a coreceptor of EMS1.

Considering the critical role of the TPD1-EMS-SERK1/2 signaling pathway in tapetum specification, it is important to uncover its downstream factors. Recently, β-CARBONIC ANHYDRASES (βCAs) and BRI EMS SUPPRESSOR 1 (BES1) family members as the downstream factors of the TPD1-EMS1-SNRK1/2 signaling pathway were identified [34,35]. In *Arabidopsis*, it is clear that βCAs play a vital role in photosynthesis through concentrating CO_2_. There are six members in the βCAs family (βCA1 to βCA6), among which βCA1, βCA2, and βCA4 have been demonstrated to interact with EMS1, suggesting that βCA1, βCA2 and βCA4 function with EMS1 in the same pathway [35,36]. Indeed, the *βca1βca2βca4* shows defects in tapetal cell differentiation and tetrad formation [35]. Moreover, βCA1, βCA2, and βCA4 can be phosphorylated by EMS1 and the phosphorylation of βCA1 leads to its activity being enhanced significantly [35]. Consistent with this observation, the phosphorylation blocking mutations of βCA1 cannot rescue the phenotype of *βca1βca2βca4*, while the phosphorylation mimic mutation is able to form tapetum [35].

**Figure 1:**
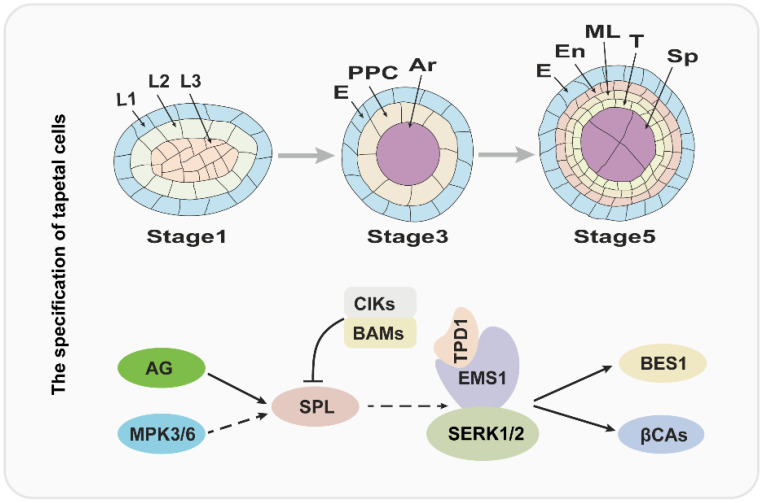
The regulatory pathway for the tapetum specification. The upper panel of the figure is a diagram of tapetum differentiation, and the lower panel is the corresponding regulatory pathway. L1, L2, L3: three cell layers of anther primordia. E: epidermis; PPC: primary parietal cell; Ar: archesporial cell. En: endothecium; ML: middle layer; T: tapetum; Sp: sporogenous cell.

The BES1 family members (BES1, BZR1, BEH1, BEH2, BEH3, BEH4), especially BES1 and BZR1, as key transcription factors, mediate an array of gene expression in the BR signaling pathway [34,37,38]. However, Chen et al. demonstrated that BES1 family members regulate tapetum development by acting as the downstream factors of the TPD1-EMS1-SERK1/2 signaling pathway [34]. The quintuple mutant, *bes1bzr1beh1beh3beh4*, fails to develop the tapetal cell layer and microspores, similar to *ems1*, *tpd1*, and *serk1/2* [34]. And the gain-of-function mutation of *BES1* or *BZR1* can partially rescue the phenotype of *ems1*, *tpd1*, and *serk1/2*. Remarkably, the expression of *TPD1* or *EMS1* driven by the *BRI1* promoter in *bri1-116*, a BR receptor knock-out mutant, significantly leads to the accumulation of non-phosphorylated, active BES1, which indicates that BES1 regulates tapetal development in a BR signaling-independent manner [34]. The molecular model is summarized in Figure 1.

## 3. The Proper Functioning of Tapetum Is Vital for Pollen Development

In angiosperms, there are two types of tapetum: the secretory and amoeboid tapetum [5,39]. The secretory tapetum is more common and widespread. The tapetum of the Cruciferae, including *Arabidopsis thaliana*, belongs to the secretory type [5]. Over the past decades, extensive studies have indicated that the tapetal cells play multiple irreplaceable functions in pollen development [5,22,23,40,41,42,43].

### 3.1. The Tapetal Cells Synthesize and Secrete Callase Complex to Degrade the Callose Wall

During the pollen development, the tapetal cells continuously provide nutrients, such as proteins lipids, and polysaccharides, for micropores from the beginning of meiosis of pollen mother cells until the complete degradation of the tapetum [5,44]. More importantly, when the pollen mother cell undergoes meiotic division to produce four microspores surrounded by a callose wall, the tapetum subsequently synthesizes and secretes callase (β-1,3-D-glucanase) complex to degrade the callose wall, leading to the release of microspores [45,46].

The callose wall is mainly composed of β-1,3-glucan [47]. Timely degradation of the callose wall is important for pollen development. Genetic studies suggested that premature or delayed callose dissolution in diffident mutants or transgenic plants leads to male sterility [48]. In *Arabidopsis*, the *Anther-specific protein6* (*A6*) is thought to be a member of the callase complex that affects callose wall degradation and is regulated by ABORTED MICROSPORES (AMS) [49,50]. The *A6* is specifically expressed in the tapetum and secreted to the anther locule after it is synthesized [49]. *AtMYB103* and *CALLOSE DEFECTIVE MICROSPORE1* (*CDM1*), which encode an R2R3 MYB protein and a tandem CCCH-type zinc finger protein, respectively, are also shown to be required for regulating the expression of *A6* [51,52]. The *ams* and *myb103* display defective tapetum, delayed callose dissolution, and abnormal exine formation [50,51]. However, the *cdm1* has no obvious tapetum development phenotype, but the callose dissolution and exine formation are impaired [52]. Recently, Wang et al. found that the *UNEVEN PATTERN OF EXINE* 1 (*UPEX1*), which encodes an arabinogalactan β-(1,3)-galactosyltransferase, plays an important role in the secretion of A6 protein [53]. The mutation of *UPEX1* does not affect the expression level of *A6*; however, the secretion of the A6 protein is delayed from tapetal cells to the locules and eventually results in a delay in callose wall degradation [53]. More interestingly, the expression of *UPEX1* is directly regulated by AMS, indicating that the AMS-UPEX1 pathway regulates the tapetum secretion function [53].

### 3.2. The Tapetum Play an Indispensable Role in the Formation of Pollen Wall

It is well known that pollen wall formation is one of the most critical steps during pollen development. The pollen wall wraps the mature pollen grains to protect the male gametophyte from numerous environmental challenges, such as high temperature, drought, and the mechanical damage caused by pathogen attacks, while also functioning in the process of pollen and stigma recognition [1,54,55]. The pollen wall is the most complex cell wall in plants, with two layers: the out layer exine and the inner layer intine [1,56]. Compared with the intine layer, the structure of the exine layer is more intricate because it is further divided into the sexine layer and nexine layer [1,56]. The sexine is composed of tectum and bacula, thus forming the complex reticulate structure of the pollen wall. The pollen coat, or tryphine, a compound containing lipids, proteins, flavonoids, and aromatic substances, fills in the interstices of the pollen exine and contributes to pollen adhesion and pollen–stigma communication [57,58]. It is clear that the tapetum plays a crucial function in the formation of the pollen wall. On the one hand, the tapetum synthesizes and secretes sporopollenin precursors, which provide the main materials for pollen exine formation [41,58]. On the other hand, the tapetal cells degenerate through programmed cell death and the degraded residues that form the pollen coat [5,59]. As a consequence, when the development of the tapetum is defective or dysfunctional, it usually leads to defects in the development of the pollen wall, which eventually causes reduced male fertility or sterility [50,60,61,62].

**Figure 2:**
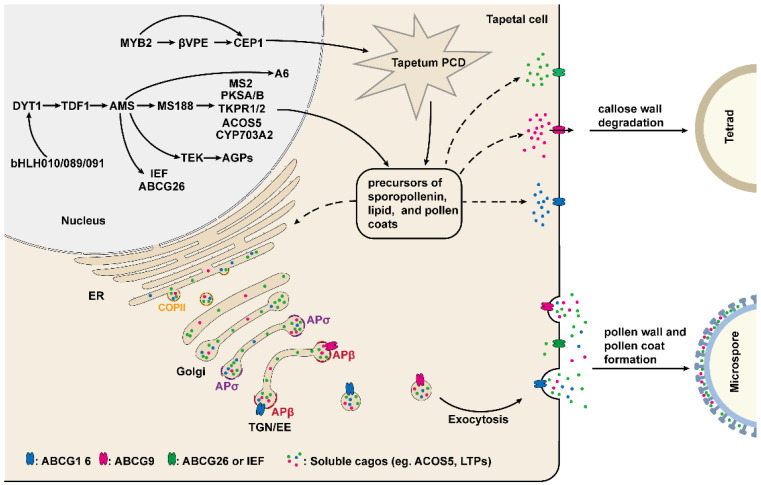
The underlying molecular mechanism of tapetum-mediated pollen development. The development and function of the tapetum are mainly regulated by a series of transcription factors. These transcription factors regulate the expression of genes required for the production of enzymes, transporters, and the precursors of sporopollenin, lipids, and pollen coats, which are indispensable for callose wall degradation, microspore development, and pollen wall and pollen coat formation. The secretion of these materials and precursors from tapetum cells to the anther locule is dependent on COPII and AP-mediated vesicle trafficking pathway.

Recent evidence indicates that the development and function of the tapetum are regulated by several key transcription factors, including DYSFUNCTIONAL TAPETUM1 (DYT1), DEFECTIVE IN TAPETAL DEVELOPMENT AND FUNCTION1 (TDF1), AMS, MALE STERILITY1 (MS1) and AtMYB103 [50,51,60,61,62]. *DYT1* encodes a putative bHLH transcription factor, which is highly expressed in the tapetum, suggesting that *DYT1* is involved in regulating the development of the tapetum [60]. Indeed, the mutation of *DYT1* leads to enlarged vacuoles in the tapetum, with microsporocytes being able to complete the first meiotic division but with abnormal cytokinesis and eventual collapse [60]. In *dyt1*, the expression of genes required for tapetum development is greatly reduced, especially for *AMS* and *MS1*, suggesting that both *AMS* and *MS1* act downstream of *DYT1* [60]. In addition, the expression level of *DYT1* is decreased in the *spl/nzz* and *ems1/exs*, implying that SPL/NZZ and EMS/EXS act upstream of DYT1 (Figure 2) [60].

Recently, three bHLH transcription factors, bHLH010, bHLH089, and bHLH091, were shown to interact with and function downstream of DYT1 [42,63]. These three genes are highly expressed in the tapetum, and their single mutants develop normally, but the various double mutants and their triple mutants exhibit an enlarged and vacuolated tapetum, delayed callose wall degradation, and aborted pollen development; which suggests that bHLH010, bHLH089, and bHLH091 function redundantly [42]. Interestingly, nuclear localization of DYT1 is closely related to the anther development stage, whereas bHLH010, bHLH089, and bHLH091 enhanced DYT1 nuclear localization by interacting with DYT1 to achieve the positive feedback regulation of *DYT1* (Figure 2) [63].

*TDF1* encodes a putative R2R3 MYB transcription factor that is critical for tapetum development, and the tapetal cells in *tdf1* exhibit aberrant cell division and dysfunction [62]. Recent studies have shown that DYT can directly bind to the promoter region of *TDF1* and in turn regulate the expression of *TDF1* [64]. The expression of *TDF1* driven by the *DYT1* promoter restored the mRNA level of tapetum development and genes required for pollen wall formation, including *AMS*, *MS1*, *MS188/MYB80*, and *TEK* in the *dyt1*, implying that DYT1 regulates these genes’ expression mainly via TDF1 [64]. Indeed, in one report, TDF1 directly regulates the expression of *AMS* via an AACCT cis-element [65]. *AMS* encodes a tapetum-specific bHLH transcription factor and is a key regulator of pollen wall formation [50,66]. Importantly, TDF1 is able to interact with AMS to form a complex that promotes the expression of AMS-regulated genes in a positive feedback manner [65]. Yeast two-hybrid assay has demonstrated that AMS can also interact with bHLH089 and BHLH091, but it is unclear what function this interaction plays [66]. In *ams*, microspore mother cells are able to undergo meiosis normally, but the tapetum becomes abnormally enlarged and vacuolated, and the tapetal cells and microspores are degraded prematurely, resulting in no pollen production in anthers [66,67]. In addition, 70 anther-specifically expressed genes are down-regulated in *ams*. Among these genes, 23 members associated with pollen wall formation are directly regulated by AMS as demonstrated by ChIP and EMSA assay [50]. Recently, several studies have shown that *MS188* (*MYB103/MYB80*), *ABCG26* (*WBC27*), and *TRANSPOSABLE ELEMENT SILENCING* VIA *AT-HOOK* (*TEK*), which play an essential role in regulating tapetum development and pollen wall formation, are directly regulated by AMS (Figure 2) [43,66,68,69,70].

MS188, a transcription factor belonging to the MYB family, is a key regulator for tapetum development, exine formation, and pollen coat deposition [51,70]. In the *ms188*, the development of tapetal cells is defective, callose dissolution is altered and pollen grains absent exine, leading to male sterile [51,70,71]. It is clear that the pollen exine is mainly composed of sporopollenin. Several genes required for sporopollenin biosynthesis, including *PKSA/B*, *MS2*, *CYP703A2*, *TKPR1/2*, *CYP704B1*, and *ACOS5*, have been identified [72,73,74,75,76,77,78]. Among these genes, *PKSA/B*, *MS2*, *CYP703A2*, and *ACOS5* are directly regulated by MS188 [72]. However, the expression of *TKPR1/2* and *CYP74B1* may depend on AMS complexed with MS188, as it has been reported that AMS and MS188 synergistically activate *CYP703A2* expression through interaction with each other [72,79].

*MS1* is another MS188 directly regulated gene which encodes a transcription factor with the PHD-finger domain and is specifically expressed in tapetum to regulate the development of tapetum and pollen [43,61,80,81,82,83]. In *ms1*, microspores have abnormal pollen exine formation, and the tapetal cells fail to undergo normal programmed cell death, eventually no pollen production in the anther [81,82,83]. Recent studies suggested that as a transcription factor, MS1 is sufficient to activate the expression of sporophytic pollen coat protein genes, including *GRP14*, *17*, *18*, *19,* and *EXL4*, *6*, which are critical for pollen–stigma interactions, pollen hydration, and environmental protection (Figure 2) [80,84,85,86,87].

Sporopollenin precursors are synthesized in tapetal cells and subsequently are transported to the pollen surface for exine formation. ABCG26 (WBC27) is an adenosine tri-phosphate binding cassette (ABC) transporter and is demonstrated to be involved in the transport of sporopollenin precursors from tapetum to anther locule [69,88,89,90]. The microspores of the *abcg26* lack normal exine, causing the degeneration of microspores at the uninucleate stage [69,90]. The expression pattern of *ABCG26* is extremely similar to the genes required for sporopollenin precursors synthesis, including *CYP704B1*, *ACOS5*, *MS2*, and *CYP703A2*, and the ABCG26 protein localizes to the plasma membrane [72,90]. Further study suggested that the substrate transported by ABCG26 is a polyketide synthesis metabolon product, the major component for forming sporopollenin [89]. Taken together, these results suggest that as a transporter of sporopollenin precursors, ABCG26 is involved in pollen sexine formation [89,90]. In addition to ABCG26, other members of the ABCG transporters family, ABCG1, ABCG9, ABCG16, and ABCG31, have also been found to be involved in the transport of pollen surface materials from tapetal cells to anther locules [91,92,93]. It has been shown in a recent report that *IMPERFECTIVE EXINE FORMATION* (*IEF*), encoding a plasma membrane protein, is another potential sporopollenin precursor transporter [94]. The *IEF* is expressed in the tapetum and is also directly regulated by AMS. Cytological observation shows both exine and nexine formation are defective in *ief* [94]. However, the expression of *ABCG26* shows no difference between wild-type and *ief* plants, and *abcg26ief-2* has a more severe sporopollenin deposition defect compared to that of *abcg26*, indicating that IEF is associated with a novel regulatory pathway for sporopollenin deposition [94]. It is likely that ABCG26 and IEF are responsible for transporting different sporopollenin constituents, which requires further investigation in the future.

*TEK* is highly expressed in the tapetal cells at the tetrad stage and encodes an AT-hook nuclear-localized family protein [68]. Cytological and genetic studies indicated that TEK determines nexine formation during pollen development and knockout *TEK* results in the absence of nexine and intine layers, while sexine formation is not affected [68]. Further study has shown that TEK directly binds to the promoter of several *Arabinogalactan proteins* (*AGPs*) genes, including *AGP6*, *AGP11*, *AGP23*, and *AGP40*, and positively regulates their expression to promote nexine formation [95]. However, in contrast to the regulation for *AGPs*, the TEK negatively regulates the expression of *CalS5* after the tetrad stage in wild-type plants, which is required for callose synthesis [47,96]. For the transgenic line of *pAMS: TEK-GFP*, the TEK expresses prematurely and suppresses *CalS5* expression directly, leading to aberrant exine patterning and defects in callose synthesis, suggesting that the temporal regulation of TEK is essential for pollen wall formation [96].

In summary, the development and function of tapetum are mainly regulated by a series of transcription factors, which constitute the genetic regulatory DYT1-TDF1-AMS-MS188/TEK/ABCG26 network to affect the pollen wall formation and pollen development through regulating the expression of different downstream genes (Figure 2) [25,97].

### 3.3. The MYB2-CEP1/βVPE Pathway Regulates the Tapetal Program Cell Death (PCD)

It is well known that tapetum PCD is one of the most important events during pollen development [4,5]. An MYB transcription factor gene family member, *MYB2*, is expressed in the tapetum from stage 5 to stage 11, and works as a key factor for the regulation of the tapetum PCD and pollen development [98]. The mutation of *MYB2* will lead to delayed tapetal PCD, irregular and spare exine, and defective pollen. The vitality and gemmation of the survival pollen in the *myb2* were greatly decreased [98]. Transcriptional activation assay showed that MYB2 functions as an activator to directly activate the expression of cysteine protease *CEP1* and *βVPE*, both of which are required for tapetal cell degradation (Figure 2) [98,99,100]. The expression pattern of *CEP1* and *βVPE* are almost overlapped with *MYB2* and similar phenotypes to *myb2* have been observed in *cep1* and *βvpe*. In addition, the *myb2* pollen vitality is rescued when either/both *CEP1* or/and *βVPE* is/are overexpressed. These results indicate that *MYB2* acts upstream and regulates the *CEP1* and *βVPE* expression for regulating tapetum PCD and pollen development [98]. However, the transcription factor that regulates the expression of *MYB2* is unclear. In tapetum-defected mutants, such as *spl/nzz*, *dyt1*, *tdf1*, *ams*, *myb80*, or *ms1*, the *MYB2* expression level has no significant change, implying that these transcription factors are not genetic upstream of MYB2 [98]. In one report, the *MYB2* expression is regulated by the transcription factor WRKY1 in response to drought and ABA treatment [101]. Furthermore, WRKY1 can bind to the W-box domain in the *MYB2* promoter, suggesting that WRKY1 acts upstream and directly regulates the expression of *MYB2* [101]. However, whether WRKY1 regulates *MYB2* expression during pollen development and the function of WRKY1 in tapetum PCD remains to be further studied.

### 3.4. The Secretory Pathway of the Tapetal Cells Is Required for Pollen Development

It is well known that the secretion of many lipids and proteins depends on vesicle trafficking after being synthesized from the endoplasmic reticulum (ER) [102]. During pollen development, the tapetal cells secrete various nutrients, enzymes, proteins, metabolites, and materials for developing microspores [5,40,41]. Therefore, the disruption of the secretory pathway usually leads to the dysfunction of the tapetum [103,104,105]. The SECRETORY31B (SEC31B) is a subunit of COPII (coat protein complex II), which mediates protein transport from the endoplasmic reticulum to Golgi and is shown to play an essential role in tapetum secretory activity [102,103]. In the *sec31b*, tapetum development is similar to the wild type, but the pollen exine formation, germination, and pollen tube growth are defective [103]. The *SEC31B* is constitutively expressed; however, the expression of *SEC31B* driven by the tapetum-specific promoter *A9* can almost completely restore the phenotypes of pollen observed in the *sec31b*, suggesting that SEC31B mainly functions in the tapetum for pollen development [103]. A recent study has revealed that other COPII components, such as SEC23A, SEC23D, and Sar1a, are also critical for tapetum development and pollen wall formation [106,107]. SEC23A and SEC23D are homologs of SEC23. The fertility of *sec23a* and *sec23d* is normal, but the exine pattern of the pollen is impaired. *sec23ad* displays more severe phenotypes, including altered tapetum development with abnormalities in organelles and delayed degradation, defects in exine and intine formation, impaired pollen coat depositions, and collapsed pollen grains [106]. The underlying molecular mechanism of SEC23A and SEC23D to regulate pollen development may be through mediating the ER transport of some essential lipids and proteins required for pollen wall formation [106]. Sar1b is one of the isoforms of Sar1. The mutation in *Sar1b* results in dysfunction of the tapetum, causing male sterility and this phenotype can be rescued by ectopic expression of *Sar1c* [107]. However, the correlated cargos mediated by the COPII complex are unclear and need to be explored in the future.

Adaptor protein complex (AP) plays an important role in clathrin-mediated vesicle formation, which has five types in *Arabidopsis*: AP-1, AP-2, AP-3, AP-4, and AP-5 [108]. The AP complex is a heterotetramer consisting of two large subunits (γ/β1, α/β2, δ/β3, ε/β4, δ/β5), a medium subunit μ and a small subunit σ [108]. Two recent studies have found that two putative AP1/2β adaptins (β1 and β2) and two AP1σ subunit proteins (AP1σ1 and AP1σ2) regulate pollen development by mediating different protein trafficking in the tapetum [105,109]. β1 and β2 are shared by the AP1 and AP2 complex. The homozygous double mutant of *β1* and *β2* cannot be obtained, and in the *β1-1β2-11*/+ and *β1-2β2-11*/+, pollen shows defects in exine formation, gemination, and pollen tube growth [109]. In the tapetal cells, β1 and β2 adaptins are nearly completely localized to the TGN, which suggests that they play a key role in TGN-dependent trafficking.

ABCG9 and ABCG16, two plasma membrane transporters, are involved in pollen surface material transport from tapetal cells to the anther locules and primarily detected in the plasma membrane in wild-type plants, while are incorrectly localized to the TGN in *β1-2 β2-11*/+ [109]. Taken together, these results indicate that in the tapetum, AP1/2β adaptins function in TGN and mediate the exocytosis of ABCG9 and ABCG16 to affect pollen development [109]. Different from AP1/2β adaptins, AP1σ1 regulates pollen development by mediating the post-Golgi trafficking of RPG1 in the microspores and the secretion of ACOS5 and type III LIPID TRANSFER PROTEINS (LTPs) from the tapetum to locules, which are associated with pollen wall formation (Figure 2) [105,110,111]. In addition, the exocytosis of ABCG9 and LTPs also can be mediated by ISTL1 and LIP5, which are two components of the endosomal sorting complex required for transport (ESCRT) and for tapetal cells function and pollen development [112].

In a report, Cui et al. discovered that the proper timing of tapetum PCD is dependent on the function of MON1/CALCIUM CAFFEINE ZINC SENSITIVITY1 (CCZ1), which activates Rab7 and thus mediates vacuolar trafficking [113]. In addition, *MON1* is highly expressed in the tapetum, and the mutation of *MON1* results in delayed tapetum PCD, which lead to delayed degradation, impaired pollen coat deposition, and male fertility [113]. Previous studies suggested that Cys proteases, which are synthesized and then transported to the vacuole for maturation, are required for the tapetal PCD [99,100]. The MON1-mediated activation of Rab7 is critical for prevacuolar compartment (PVC)-to-vacuole trafficking. However, in *mon1*, the Cys protease RD21 exhibits abnormal accumulation in enlarged PVC. Collectively, these results suggest that MON1–Rab7 complex-mediated vacuolar trafficking is essential for tapetal PCD and pollen development [113].

## 4. Conclusions and Perspectives

In higher plants, pollen development is an extremely complex process, which is regulated not only by gametophytic but also sporophytic factors, especially the tapetum for the latter case [2,5,12]. The tapetum is a monolayer of cells surrounding the microsporocyte/microspores that provides various nutrients, enzymes, proteins, and lipids for the developing male gametophyte [5]. Over the past two decades, the underlying molecular mechanism of tapetum-mediated pollen development has been gradually elucidated. In this review, we summarize the recent advance in tapetum-mediated pollen development, and draw the following conclusions: Firstly, the differentiation of tapetum is important for pollen development and regulated by the TPD-EMS1-SERK1/2-βCAs/BES1 pathway, with associated gene mutations resulting in a lack of tapetum and excess microsporocytes. Secondly, a number of transcription factors play a major role in regulating the tapetum’s development and function. These transcription factors constitute the DYT1-TDF1-AMS-MS188/TEK transcriptional regulatory network and regulate pollen development by mediating the expression of different downstream genes. In addition, the MYB2-CEP1/βVPE pathway mediated tapetal PCD, which is independent of the DYT1-TDF1-AMS-MS188/TEK transcriptional regulatory network. Finally, the secretion of enzymes, transporters, and pollen precursors by tapetum for pollen development is likely to depend on COPII and AP-mediated vesicle trafficking pathways.

Although the underlying mechanisms of tapetum-mediated pollen development are well understood, many questions remain to be addressed, for example, how the signal communication between the microspores and tapetum takes place; what are the signals that induce tapetal PCD and how; and which transporters mediate the transports of the materials secreted by the tapetum to the pollen surface.
